# The Dual Burden of Weight in YMSM: Structural Inequities and Body Image Pressures

**DOI:** 10.1007/s40615-026-03037-4

**Published:** 2026-06-15

**Authors:** Ching-Yu Wang, Dustin T. Duncan, Lisa M. Kuhns, Robert Garofalo, Jean Jimenez, Patrick R. Veihman, Rebecca Schnall

**Affiliations:** 1 School of Nursing, Columbia University, New York, NY 10032, United States of America; 2 School of Nursing, University of North Carolina at Greensboro, Greensboro, NC 27403, United States of America; 3 Mailman School of Public Health, Department of Epidemiology, Columbia University, New York, NY 10032, United States of America; 4 Division of Adolescent & Young Adult Medicine, Ann & Robert H. Lurie Children’s Hospital of Chicago, 225 East Chicago Avenue, Chicago, IL 60611, United States of America; 5 Feinberg School of Medicine, Northwestern University, Chicago, IL 60611, United States of America

## Abstract

**Background/Objectives:**

Young men who have sex with men (YMSM) navigate a unique intersection of health risks characterized by structural drivers of obesity and intense sociocultural pressures for thinness. While Body Mass Index (BMI) differences are documented in the general population, less is known about how these patterns manifest within the specific appearance-potent subcultures of YMSM. This study describes BMI profiles across racial/ethnic groups in a large YMSM cohort to examine the interplay between weight status and the dual pressures of structural inequity and body image disturbance.

**Methods:**

We conducted a cross-sectional analysis using data from 2,704 YMSM (age 17–29 years) in a US digital cohort study. Participants self-reported height, weight, race, and ethnicity. We used Analysis of Variance (ANOVA) and Chi-squared tests to assess BMI differences across five racial/ethnic groups (Asian Non-Hispanic, Black Non-Hispanic, Hispanic, White Non-Hispanic, Other Non-Hispanic).

**Results:**

Significant variations in mean BMI (F(4, 2699) = 13.59, *p* < 0.001) and weight category distribution (χ^2^(12) = 73.05, *p* < 0.001) were observed. Asian Non-Hispanic and White Non-Hispanic men exhibited significantly lower mean BMIs and higher prevalence of healthy weight classification compared to Black and Hispanic peers. However, in the context of YMSM culture, this lower weight profile warrants clinical attention, as prior literature suggests it may co-occur with restrictive behaviors and body image disturbance. Conversely, Black Non-Hispanic and Hispanic men exhibited higher mean BMIs, reflecting persistent structural disparities.

**Conclusions:**

Significant racial and ethnic differences in BMI exist among US YMSM, reflecting a polarized risk environment. The BMI profiles of Black and Hispanic YMSM are consistent with structural risk patterns documented in the broader population, while the lower BMI profiles of White and Asian YMSM align with prior literature on body image pressures in gay subcultures. Both structural and sociocultural risks are relevant across all groups. These findings suggest that weight status in YMSM serves as a marker for divergent sociocultural and structural pressures. Public health strategies must address this dual burden by targeting both obesogenic environments and toxic body ideals.

## Introduction

Weight status represents a complex public health challenge in the United States (US). While the prevalence of overweight and obesity drives significant risk for chronic conditions like cardiovascular disease and type 2 diabetes in the general population [1, 2], weight-related health risks manifest differently within specific subpopulations. For young men who have sex with men (YMSM), weight status is not solely defined by the risk of obesity but is influenced by a unique constellation of pressures including minority stress, sexual identity stigma, and intense sociocultural scrutiny regarding body image [3, 4]. YMSM occupy appearance-potent subcultures that perpetuate strict body standards and emphasize a paradoxical ideal of being both thin and muscular [5, 6]. Consequently, examining Body Mass Index (BMI) in this population requires a lens that captures the full spectrum of weight status, as health risks may stem from both obesity-related comorbidities and the weight-restrictive behaviors associated with the drive for thinness [5, 7].

These weight-related pressures do not occur in isolation but intersect with race and ethnicity to create a dual burden of health risk. On one hand, YMSM are exposed to community norms that valorize leanness, which recent systematic reviews link to higher rates of body image disturbance and restrictive eating compared to heterosexual men [6]. This pressure can manifest as lower BMI profiles that mask pathological weight control behaviors under the guise of healthy weight. On the other hand, YMSM of color must navigate these community pressures alongside structural inequities found in the general population, such as food apartheid, limited access to safe recreational spaces, and socioeconomic stratification [8, 9]. This intersection creates a complex landscape where racial and ethnic minority men may face compounded risks. They experience the general societal drivers of obesity while simultaneously facing marginalization within gay subcultures for not adhering to the prevailing thin body standards.

Current literature often focuses on obesity disparities in isolation, potentially overlooking the nuances of this dual burden. Looking solely at overweight prevalence misses the significant proportion of YMSM who may be underweight or maintaining low weight through unhealthy means. Furthermore, reliance on general population BMI trends may obscure how the specific body image pressures of the gay community alter weight profiles across different racial and ethnic groups. For instance, do YMSM of color experience the same structural drivers of obesity as their heterosexual peers, or do the unique body image pressures of the gay community alter these profiles? Investigating these variations is critical, as clinical interventions must distinguish between weight status driven by structural deprivation and weight status driven by body dysmorphia.

The primary objective of this study was to examine variations in BMI profiles across five racial and ethnic groups (Asian Non-Hispanic, Black Non-Hispanic, Hispanic, White Non-Hispanic, and Other Non-Hispanic) within a large, geographically diverse sample of YMSM. By analyzing both mean BMI and the distribution across weight categories, including underweight and healthy weight, we aim to provide a nuanced understanding of how race, ethnicity, and sexual identity intersect to shape weight status. We posit that weight status in this population serves as a marker for divergent pressures, reflecting structural inequities for some subgroups and intense sociocultural body image demands for others.

## Methods

### Study Population and Recruitment

Data for this secondary analysis were drawn from a parent study investigating Human Immunodeficiency Virus (HIV) incidence among young men who have sex with men (YMSM) in the United States and its territories [10]. Participants in the parent study completed surveys online via Research Electronic Data Capture (REDCap) after providing electronic informed consent. The parent study protocol was reviewed and approved by the Columbia University Institutional Review Board (Protocol #AAAU2559), which served as the IRB of record for all participating institutions [10]. Only de-identified data were utilized for the analyses presented in this paper.

The initial dataset comprised 3,009 participants. Participants identified as Body Mass Index (BMI) outliers using the standard Interquartile Range (IQR) method (values below Q1–1.5 * IQR or above Q3 + 1.5 * IQR) were first excluded. Subsequently, participants with missing self-reported height or weight data (required for BMI calculation) or missing race/ethnicity data were also excluded. This resulted in a final analytical sample of 2,704 individuals with valid data for all variables included in the primary analyses.

## Measures

### Body Mass Index (BMI)

BMI was calculated as weight in kilograms divided by height in meters squared (kg/m^2^), based on self-reported height and weight. BMI was analyzed as a continuous variable and was also categorized using standard World Health Organization (WHO) thresholds: Underweight (< 18.5 kg/m^2^), Healthy Weight (18.5–24.9 kg/m^2^), Overweight (25.0–29.9 kg/m^2^), and Obese (≥ 30.0 kg/m^2^). We acknowledge the clinical relevance of population-specific BMI thresholds, particularly evidence supporting lower cutoffs (e.g., ≥ 23 kg/m^2^) for defining overweight in Asian populations to better capture metabolic risk [11]. However, for the purposes of this study, standard WHO international classifications were utilized to maintain direct comparability with the parent study protocols and national reference data for the general US population. Consequently, the use of standard thresholds may result in conservative estimates of weight-related risk for Asian Non-Hispanic participants. This limitation is considered in our interpretation of healthy weight as a category that may obscure metabolic risk for some subgroups while reflecting sociocultural pressures for others.

### Race/Ethnicity

Participants’ race and ethnicity were self-identified through a two-step question sequence within the online REDCap survey, consistent with common US demographic data collection practices. Participants first indicated their Hispanic ethnicity by selecting from options including “No, not Hispanic/Latino”, “Yes, Cuban”, “Yes, Dominican”, “Yes, Mexican/Mexican American”, “Yes, Puerto Rican”, or “Yes, other”. Subsequently, participants were asked to select one or more racial categories from the following: “American Indian or Alaskan Native”, “Asian/Asian American”, “Black/African American”, “Native Hawaiian or Other Pacific Islander”, “White/Caucasian”, “Multiracial”, or “Something else (please specify)”. Based on responses to these two questions, participants were classified into five mutually exclusive analytical categories: (a) Hispanic: endorsed any “Yes” option for Hispanic ethnicity; (b) White Non-Hispanic: endorsed “No, not Hispanic/Latino” and selected only “White/Caucasian” race; (c) Black Non-Hispanic: endorsed “No, not Hispanic/Latino” and selected only “Black/African American” race; (d) Asian Non-Hispanic: endorsed “No, not Hispanic/Latino” and selected only “Asian/Asian American” race; (e) Other Non-Hispanic: endorsed “No, not Hispanic/Latino” and selected “American Indian or Alaskan Native”, “Native Hawaiian or Other Pacific Islander”, “Multiracial”, “Something else”, or more than one racial category.

### Statistical Analysis

Descriptive statistics, including means, standard deviations (SD), frequencies, and percentages, were computed for BMI (continuous and categorical) and participant demographics (race/ethnicity) using the final analytical sample (*N* = 2,704). Analysis of Variance (ANOVA) was employed to assess overall differences in mean BMI across the five racial/ethnic groups. Following a significant ANOVA result, post-hoc pairwise comparisons were conducted using Tukey’s Honestly Significant Difference (HSD) test to identify specific between-group differences. A Pearson’s Chi-squared test was utilized to examine the association between race/ethnicity and the distribution of participants across the four BMI categories. All analyses were performed on the final sample of *N* = 2,704. All statistical analyses were performed using R version 4.3.3 [12] within the RStudio environment (version 2024.04.2 + 764; [13]). Statistical significance was established a priori at *p* < 0.05.

## Results

### Sample Characteristics

The final analytical sample comprised 2,704 participants after exclusions for outlier BMI values, missing BMI data, and missing race/ethnicity data. The racial and ethnic composition and BMI characteristics of this sample are detailed in Table 1. The overall sample included Asian Non-Hispanic (*N* = 211), Black Non-Hispanic (*N* = 419), Hispanic (*N* = 819), Other Non-Hispanic (*N* = 68), and White Non-Hispanic (*N* = 1187) individuals. The mean BMI for this sample (*N* = 2,704) was 25.52 kg/m^2^ (SD = 5.18). Notably, the distribution was skewed toward the lower end of the weight spectrum compared to general population trends, with the largest proportion of participants falling within the healthy weight category (48.9%; *n* = 1321), followed by Overweight (28.3%; *n* = 766), Obese (18.9%; *n* = 510), and Underweight (4.0%; *n* = 107).

### Mean BMI Differences by Race/Ethnicity

**Figure 1:**
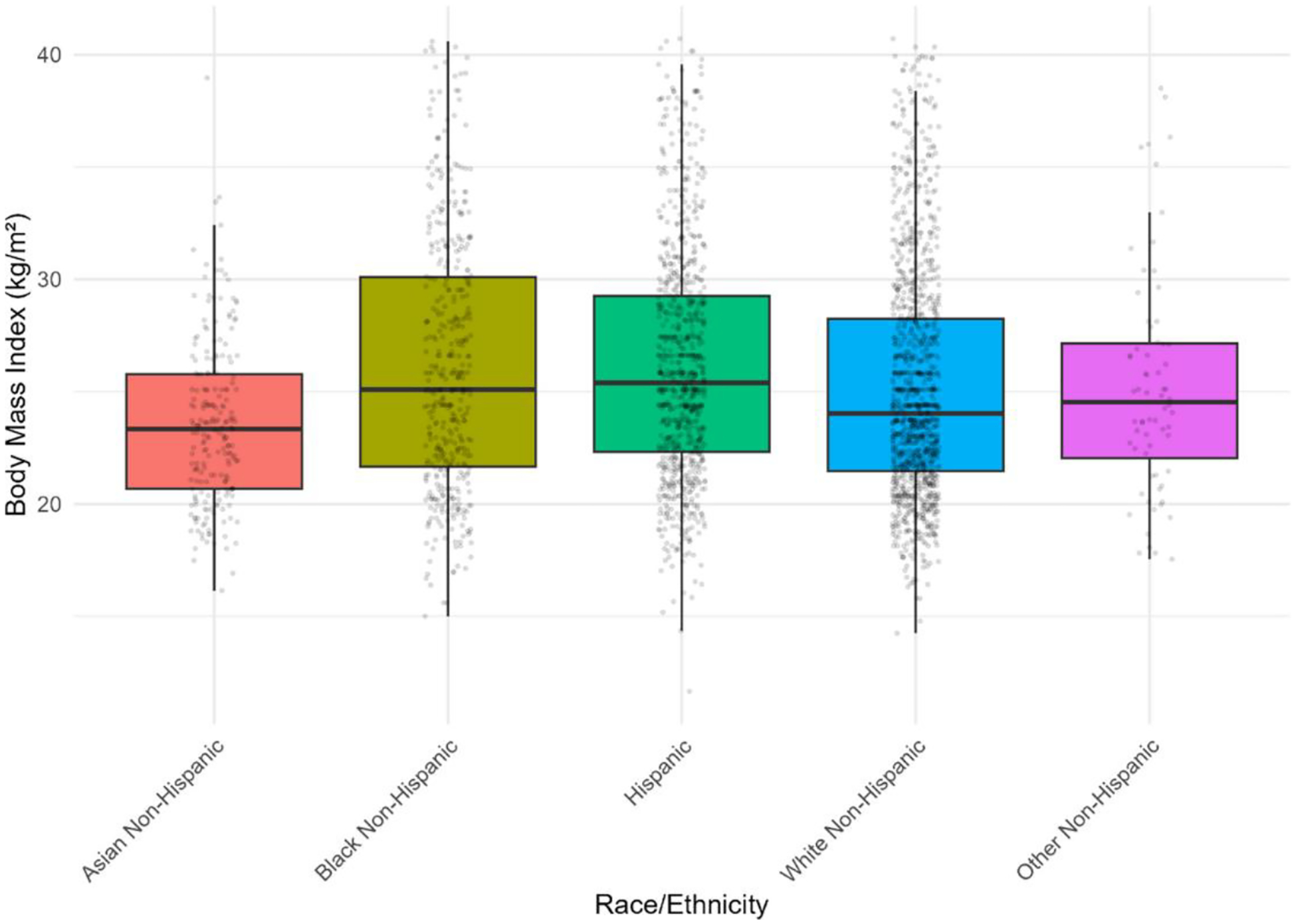
Distribution of BMI by Race/Ethnicity among YMSM

Analysis of variance revealed a statistically significant difference in mean BMI across the five racial/ethnic groups (F(4, 2699) = 13.59, *p* < 0.001). Group-specific mean BMI values were as follows: Asian Non-Hispanic (M = 23.56, SD = 3.61), Black Non-Hispanic (M = 26.14, SD = 5.70), Hispanic (M = 26.17, SD = 5.17), Other Non-Hispanic (M = 25.27, SD = 5.01), and White Non-Hispanic (M = 25.22, SD = 5.13). Post-hoc analyses using Tukey’s HSD test identified several statistically significant pairwise variations. Asian Non-Hispanic men had significantly lower mean BMI compared to Black Non-Hispanic men (mean difference = 2.59, *p* < 0.001), Hispanic men (mean difference = 2.61, *p* < 0.001), and White Non-Hispanic men (mean difference = 1.67, *p* < 0.001). Additionally, White Non-Hispanic men exhibited lower mean BMI than both Black Non-Hispanic (mean difference = −0.92, *p* = 0.014) and Hispanic men (mean difference = −0.94, *p* < 0.001). No statistically significant differences were observed between Black Non-Hispanic and Hispanic men. The distribution of BMI showed in Figure 1.

### Association Between BMI Category and Race/Ethnicity

**Figure 2:**
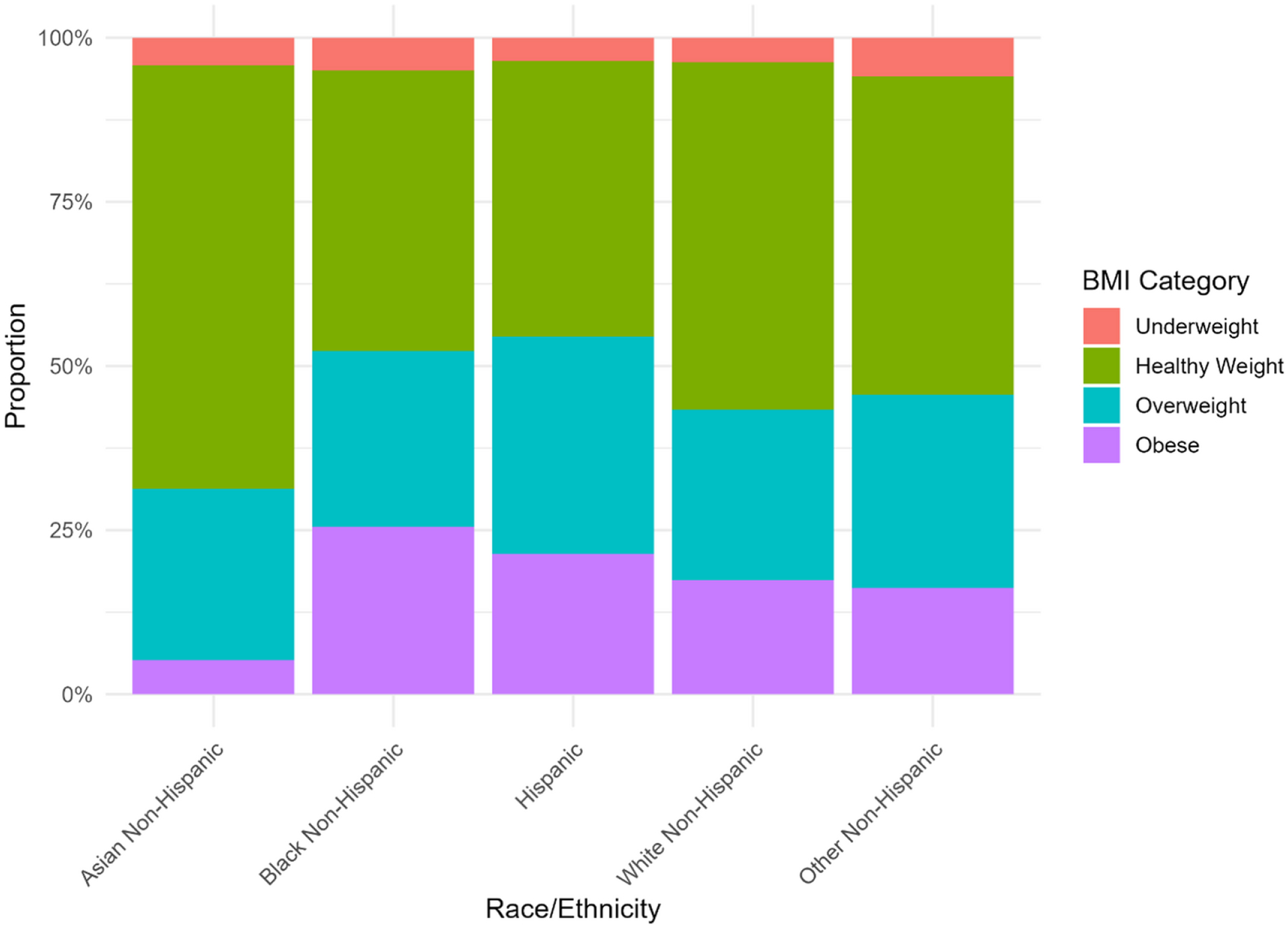
Proportion of BMI Categories by Race/Ethnicity among YMSM

Pearson’s Chi-squared test demonstrated a statistically significant association between race/ethnicity and BMI category distribution (χ^2^(12) = 73.05, *p* < 0.001). As shown in Figure 2, the cohort is characterized by a polarized distribution of weight status. Asian Non-Hispanic men exhibited the highest prevalence of Healthy Weight (64.5%), followed by White Non-Hispanic men (53.0%). In contrast, Black Non-Hispanic (25.5% Obese) and Hispanic men (21.4% Obese) had higher representation in the Obese category relative to their Asian Non-Hispanic (5.2% Obese) and White Non-Hispanic (17.4% Obese) counterparts.

## Discussion

In this large, national sample of YMSM, the combined prevalence of overweight (28.3%) and obesity (18.9%) was 47.2%. While substantial, this value is notably lower than recent national estimates for the general population of US men in a similar age range (72.3%) [14]. Consequently, the prevalence of healthy weight in our sample (48.9%) was considerably higher than in the general population. While traditionally interpreted solely as a positive health indicator, this lower weight profile must be viewed through a dual lens. It may reflect younger age and resilience, but it also likely reflects the unique sociocultural pressures of the YMSM community where a strong drive for thinness often coexists with the desire for muscularity [5, 6].

This study identified significant racial and ethnic differences in BMI profiles. The observed patterns, wherein Asian Non-Hispanic YMSM had the lowest mean BMI and highest proportion with healthy weight, while Black Non-Hispanic and Hispanic YMSM exhibited higher mean BMIs, and White Non-Hispanic YMSM held an intermediate position, largely mirror general population trends [15] but require specific contextualization for YMSM. The significantly lower BMI among Asian and White YMSM in our sample aligns with recent systematic reviews indicating that sexual minority men are often more prone to body image disturbance and restrictive eating behaviors than heterosexual men [6]. As noted by Schmidt et al. (2022), gay men frequently navigate a conflicting body ideal that demands one be both lean and muscular [5]. This subculture is characterized by a potent focus on appearance that can drive behaviors resulting in lower BMI, potentially masking disordered eating or unhealthy weight control behaviors under the guise of a clinically healthy weight [6]. Therefore, the lower BMI observed in these subgroups should not be interpreted purely as an absence of cardiovascular risk. Prior literature suggests that lower BMI in YMSM may co-occur with body image disturbance and restrictive behaviors, warranting clinical attention even when BMI falls within a healthy range.

Conversely, the higher mean BMI profiles observed among Black Non-Hispanic and Hispanic YMSM are consistent with patterns documented in the general population, where structural inequities, such as limited access to nutritious food, reduced access to safe recreational spaces, and socioeconomic stratification, have been associated with elevated weight status [8, 9]. While this study did not directly measure these structural factors, the observed BMI distributions align with established population-level evidence suggesting that such inequities may shape weight status within this subgroup, potentially interacting with or overriding community pressure to be thin. Future research should directly examine these structural determinants within YMSM samples. Minority stress theories offer one framework for understanding these patterns, positing that the compounded burden of racism and homophobia may exacerbate stress-related health behaviors or reduce resources available for health-promoting activities [6]. While this study did not measure minority stress directly, this theoretical lens may help contextualize the observed BMI distributions and warrants examination in future research. It is crucial to note that these men may face a dual compounding risk characterized by stigmatization within the broader society for their race and sexual orientation, and further marginalization within the gay community for not adhering to the prevailing body standards.

Furthermore, interpretation of these findings must account for the inherent limitations of Body Mass Index (BMI) as a metric. Recent scientific statements from the American Heart Association emphasize that BMI often fails to capture metabolic risk, noting that excess visceral adiposity (belly fat) raises the risk of cardiovascular disease even among individuals classified as having a healthy BMI [16]. This limitation is critical as BMI does not distinguish between body fat and lean muscle mass, nor does it account for racial or ethnic differences in body composition. For example, evidence supports using lower BMI thresholds (≥ 23 kg/m^2^) to identify metabolic risk in Asian populations [11], meaning the lower BMI of Asian YMSM in our sample may still carry health risks. Conversely, BMI may overestimate adiposity in individuals with higher muscle mass. Therefore, the differences observed here should be viewed as variations in weight status distributions rather than definitive measures of health disparities in isolation.

These findings underscore the necessity of applying an intersectional lens to YMSM health. BMI is shaped by the convergence of biological, structural, and sociocultural forces [8]. Interventions must be nuanced and intersectional, recognizing that structural and clinical risks are present across all racial and ethnic groups, though their relative salience may differ. For Black and Hispanic YMSM, whose BMI profiles mirror patterns associated with structural inequities in the broader population, strategies that address barriers to healthy eating and physical activity may carry particular importance. At the same time, clinical vigilance regarding disordered eating and body dysmorphia is essential across all subgroups, including Black and Hispanic men who simultaneously navigate community body ideals alongside structural pressures. For YMSM with lower BMI profiles, particularly within White and Asian subgroups, this clinical awareness is especially urgent, as a healthy-range BMI may obscure pathological weight control behaviors [5]. No group is fully insulated from either structural risks or clinical concerns, and tailored approaches should account for this complexity. weight is not maintained through pathological restriction or body dysmorphia [5].

This study’s strengths include its large sample size (*N* = 2,704 with complete data) and diverse racial/ethnic composition, facilitated by a national online recruitment strategy. However, several limitations warrant consideration. First, reliance on self-reported height and weight introduces potential measurement bias; studies, particularly among women, indicate individuals tend to overestimate height and underestimate weight, which can lead to clinically significant errors in BMI classification [17]. The magnitude and direction of this bias can vary; for example, height overestimation may be more pronounced in men, shorter individuals, and older adults, while weight underestimation tends to increase with actual weight [18]. Although our sample consists of men, the potential for such self-report biases underscores that direct measurement is preferable when feasible [17, 18]. Second, the cross-sectional design prohibits causal inference and the examination of BMI changes over time. Third, the analysis did not include detailed data on potential confounders such as dietary intake, physical activity, socioeconomic indicators, or acculturation, which could explain how or why the observed associations occur or identify specific subgroups for whom these associations are stronger, as delineated by Baron and Kenny [19]. Fourth, the predominantly web-based recruitment may affect generalizability, a priori excluding YMSM with limited internet access or yielding a sample that differs systematically from those recruited through venue-based or other methods. Fifth, the small size (*n* = 68) and heterogeneity of the Other Non-Hispanic group restricted meaningful analysis and interpretation for this population segment. Finally, participants excluded prior to analysis due to outlier BMI, missing BMI, or missing race/ethnicity might differ systematically from the final analytical sample, potentially introducing bias if the reasons for exclusion were related to participant characteristics or outcomes.

## Conclusion

This study documented significant racial and ethnic variations in BMI within a large, national sample of young YMSM (*N* = 2,704) in the United States. The observed polarized patterns, with Asian and White men exhibiting lower BMI profiles and Black and Hispanic men exhibiting higher profiles, reflect the dual burden of risk in this population. For some, weight status may be associated with the internalized pressure for leanness that prior literature identifies as pervasive in YMSM subcultures; for others, BMI profiles are consistent with patterns linked to structural inequities in the broader population. Importantly, these forces are not mutually exclusive, and all subgroups may experience both to varying degrees. These findings underscore the critical need for public health interventions to adopt an intersectional perspective, acknowledging that weight management for YMSM requires addressing both the structural roots of obesity and the toxic sociocultural pressures of body image disturbance.

## Figures and Tables

**Table 1:** Baseline Characteristics of YMSM Sample by Race/Ethnicity (*N* = 2704)

Characteristic,	Overall sample (*N* = 2704)	Asian non-hispanic (*N* = 211)	Black non-hispanic (*N* = 419)	Hispanic (*N* = 819)	Other non-hispanic (*N* = 68)	White non-hispanic (*N* = 1187)	*p*-value
BMI (kg/m^2^), Mean (SD)	25.52 (5.18)	23.56 (3.61)	26.14 (5.70)	26.17 (5.17)	25.27 (5.01)	25.22 (5.13)	< 0.001^1^
BMI Category, n (%)							< 0.001^2^
Underweight (< 18.5)	107 (4.0)	9 (4.3)	21 (5.0)	29 (3.5)	4 (5.9)	44 (3.7)	
Healthy (18.5–24.9)	1321 (48.9)	136 (64.5)	179 (42.7)	344 (42.0)	33 (48.5)	629 (53.0)	
Overweight (25–29.9)	766 (28.3)	55 (26.1)	112 (26.7)	271 (33.1)	20 (29.4)	308 (25.9)	
Obese (≥ 30)	510 (18.9)	11 (5.2)	107 (25.5)	175 (21.4)	11 (16.2)	206 (17.4)	

*Abbreviations:* SD, Standard Deviation; BMI, Body Mass Index; YMSM, Young Men who have Sex with Men

Table includes participants from the parent study with valid BMI data and non-missing race/ethnicity (*N* = 2704). An initial *N* = 3009 had participants excluded due to outlier BMI values, missing BMI data, or missing race/ethnicity data.

Percentages calculated based on column totals (Overall *N* = 2704 or Group N).

1 p-value from ANOVA test comparing mean BMI across groups.

2 p-value from Pearson’s Chi-squared test comparing distribution of BMI categories across groups.

## Data Availability

Deidentified individual data and a data dictionary will be made available upon reasonable request after the approval of a proposal and signing of a data use agreement.
